# Residue of Picloram in New Agricultural Areas: Impacts, Detection, and Mitigation Measures

**DOI:** 10.1021/acsomega.6c02204

**Published:** 2026-05-04

**Authors:** Guilherme Braga Pereira Braz, Matheus de Freitas Souza, Dieimisson Paulo Almeida, Camila Jorge Bernabé Ferreira, Rafael da Silva Cabral, Éder Vaz de Almeida, Sergio de Oliveira Procópio

**Affiliations:** † 245072 Universidade de Rio Verde, Rio Verde, Goiás 75901-970, Brazil; ‡ Syngenta, Formosa, Goiás 73801-970, Brazil; § EMBRAPA Meio Ambiente, Jaguariúna, São Paulo 13918-110, Brazil

## Abstract

The expansion of grain production into former pasture areas in Brazil, one of the biggest grain producers’ countries worldwide, has intensified concerns regarding the residual effects of auxinic herbicides historically used for broadleaf weed control. Among these, picloram (4-amino-3,5,6-trichloropicolinic acid) stands out due to its high persistence and mobility in soil, posing a significant risk to sensitive rotational crops. This review discusses the implications of picloram use in pasture systems and its impact on the incorporation of these areas into soybean-based grain production systems. Owing to its low adsorption coefficient and moderate water solubility, picloram exhibits considerable leaching potential and a prolonged half-life, which may extend beyond 300 days depending on soil and climatic conditions. Its primary dissipation pathway occurs via aerobic microbial degradation, and reduced oxygen availability at deeper soil layers can further prolong its residual activity. Soybean, dry beans, and tomato are highly sensitive to picloram residues, with phytotoxic symptoms including epinasty, leaf deformation, root thickening, growth inhibition, and yield reduction. Even low soil concentrations have been associated with significant grain yield losses, characterizing a carryover effect that may persist for more than two growing seasons. Additionally, the herbicide may remain biologically active after passage through livestock digestive systems, raising concerns regarding manure contamination. Detection strategies include herbicide application history analysis, bioassays using sensitive indicator species, and chromatographic techniques, each presenting methodological limitation when used individually. Mitigation approaches include rational herbicide use, avoidance of picloram in areas intended for future grain production, phytoremediation strategies, and crop rotation with more tolerant species such as corn and sorghum. Given the projected increase in soybean cultivation in areas formerly under pasture, understanding picloram behavior in soil and its agronomic implications is essential to ensure sustainable land–use transition and prevent yield losses associated with herbicide carryover.

## Graphical abstract

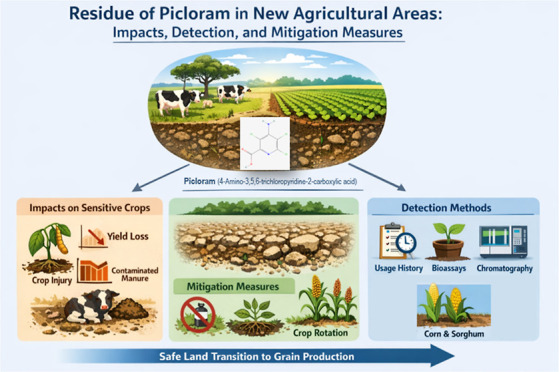

## 1 Use of Picloram and the Incorporation of Areas for Grain Production

Analogous to what happens in agricultural production systems, when weeds infest pasture areas they end up causing serious limitations for livestock activity, leading to lower biomass production, decreased nutritional quality of forage, reduced carrying capacity of the pasture, animal poisoning due to the presence of toxic species, among other losses resulting from the process of interference.^1^ In this context, the need to adopt measures for the integrated management of weeds in pasture areas becomes evident, with chemical control, carried out through the application of herbicides, being one of the most widely used methods in these environments.

Regarding the chemical control of weeds in pasture areas, one of the identified challenges concerns the management of species classified as narrow-leaved (monocotyledons), since their morphological similarity to forage limits the use of herbicides for effective and selective control.^2^ On the other hand, for the control of broad-leaved weeds (dicotyledons), there are several herbicides registered for use in pastures, which generally show good efficacy when applied at the recommended doses, methods, and stages.

Among the herbicides widely used to control broadleaf weeds in pasture areas, several contain active ingredients whose mechanism of action involves auxin mimicking (HRAC and WSSA: 4), also known as hormonal or auxinic herbicides. These herbicides are registered for controlling both herbaceous and shrubby dicotyledonous weeds (featuring some active ingredients with a spectrum against monocotyledons, for example florpyrauxifen) and can be applied either as broadcast (total area) or as spot treatments (foliar, cut stump, or basal applications). In Brazil, there are different auxinic herbicides registered for use in pastures, belonging to four distinct chemical groups within this mechanism of action. Among the most common active ingredients are 2,4-D amine, fluoroxypyr, triclopyr, aminopyralid, picloram, and aminocyclopyrachlor (Figure 1). There are also other auxinic herbicides registered for use in Brazil that belong to different chemical groups within this mechanism of action but are not commonly used in pasture areas (benzoates, quinoline-carboxylates, 6-arylpicolinates, and others such as benazolin-ethyl).

**Figure 1:**
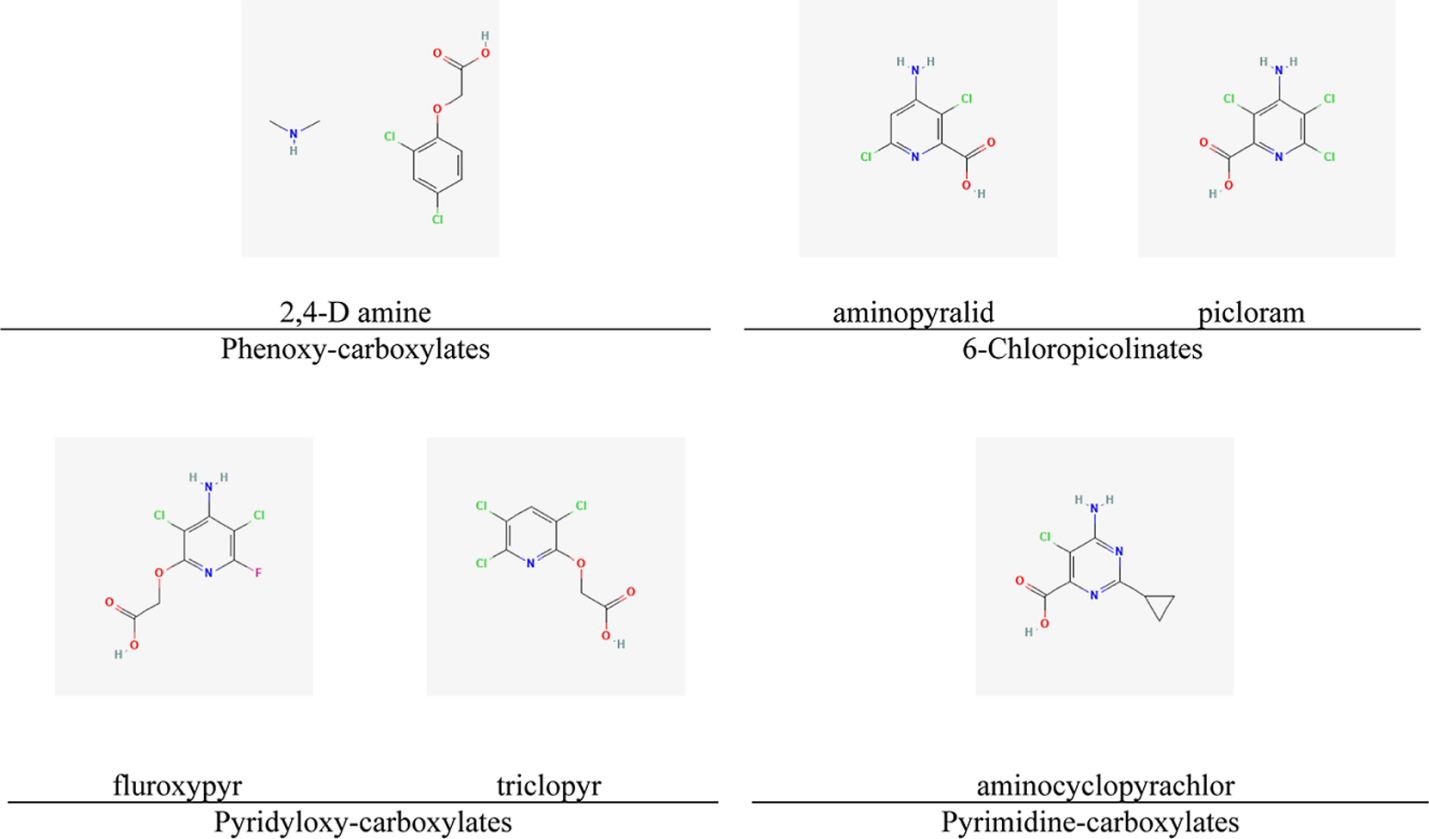
Chemical groups and molecular structures of the active ingredients of auxinic herbicides.

However, it is important to highlight that one of the main drawbacks regarding the use of these herbicides is the long residual activity that certain active ingredients may exhibit, as in the cases of picloram and aminopyralid, which have been reported to have residual effects in the soil on crops for more than two years after application.^3^ The duration of the residual period of these herbicides depends on the applied dose and the physicochemical properties of the soil environment.^4^ For areas used for livestock activities where there is no intention to shift to agriculture, the long residual period in the soil presented by herbicides such as picloram and aminopyralid does not constitute a serious obstacle. However, within the process of the expansion of Brazilian agriculture, especially in the *Cerrado* biome, a significant portion of the areas to be incorporated will come from locations with a history of pasture cultivation. In these environments, the residual effect of hormonal herbicides will be a serious bottleneck for the production system, as they can render the cultivation of certain crops, such as soybeans, beans, cotton, and tomatoes, unfeasible,^5,6^ a phenomenon known as carryover.

Among the crops occupying areas previously used for livestock farming, soybeans stand out the most. The cultivation of soybeans in underutilized pasture areas in the Brazilian *Cerrado* has been considered an important strategy to curb the deforestation of areas with native vegetation. According to The Nature Conservancy (TNC), in 2016/2017, about 38% of soybean production came from areas covered by native vegetation.^7^ To address this situation, the authors suggest an investment policy aimed at converting pasture areas, often in an advanced state of degradation, into areas used for grain cultivation, mainly soybeans.

This approach has been encouraged among rural producers, already resulting in effects in the 2023/24 Brazilian agricultural season. According to the Companhia Nacional de Abastecimento (CONAB), the estimated grain harvest for 2025/26 is expected to increase by 5% due to the expansion of the soybean planted area from 47,3 to 49,1 million hectares.^8^ Most of this increase in planted area will be the result of occupying areas currently under pasture systems. Despite the potential for using pasture areas, especially degraded ones, to increase grain production, there is a limitation imposed by the herbicides picloram and aminopyralid for soybean cultivation. As mentioned earlier, soybeans are extremely sensitive to the presence of these herbicides in the soil. Consequently, the high persistence of picloram and aminopyralid can limit the adoption of this oilseed crop due to the presence of residues at levels sufficient to reduce crop yield.

## 2 Physicochemical Properties of Picloram and Its Behavior in Soil

Picloram is a systemic herbicide belonging to the 6-chloropicolinates acid chemical group, registered for the management of weeds in pasture areas and sugar cane crops. Additionally, some commercial products are registered for the eradication of eucalyptus stumps. Currently, there are more than 60 commercial products containing this active ingredient in Brazil, available in formulations with picloram alone or in double mixtures with 2,4-D, fluoroxypyr, or triclopyr, as well as triple mixtures with aminopyralid + fluoroxypyr, aminopyralid + triclopyr, and fluoroxypyr + triclopyr. As previously mentioned, the use of commercial products based on picloram is aimed at controlling broadleaf weeds with herbaceous or shrubby vegetative habits. It is important to point out that in Brazil picloram is marketed in five types of conjugates, namely: picloram dimethylamine salt, picloram triethanolamine salt, picloram triisopropanolamine salt, picloram potassium salt, and picloram hexylpropylamine salt.^9^ No picloram ester is currently marketed in Brazil, such as, for example, picloram isooctyl ester.

Regarding the behavior of picloram in the soil environment, due to its low adsorption coefficient (*k*_f_ = 0.46) for most soils and moderate water solubility (430 mg L^–1^), this herbicide has a high potential for leaching, and it is common to find residues at depths of up to 60 cm from the soil surface.^10^ Although it shows weak adsorption to certain types of clay, increasing organic matter content can partly promote the adsorption process of the herbicide in the soil.

Picloram degradation in the soil is relatively slow compared with that of other herbicides, contributing to its greater persistence in the environment. Under field conditions, its half-life is approximately 90 days, although it may extend to up to 300 days depending on soil and climatic conditions. The main dissipation pathway of this herbicide occurs through metabolism by aerobic microorganisms.^11^ In addition, its relatively stable chemical structure contributes to a slower degradation process compared with herbicides that are more readily metabolized by soil microbial communities. Due to its mobility in the soil profile, picloram may reach depths of up to 60 cm, which can prolong its residual period, since reduced soil oxygenation at greater depths considerably limits microbial degradation activity.^12^

Due to the long period required for this molecule to dissipate 50% of the initially applied concentration in the soil, combined with the high susceptibility of soybeans to the presence of picloram residues, these factors together make it common to receive reports of crop injury even more than two years after the herbicide had been applied.^10,13^ In addition to the extended residual period of this herbicide, the sensitivity of crops such as soybeans, beans, and tomatoes to auxin mimics^4,5,14^ increases the risk of intoxication in areas where picloram has been used. Other aggravating factors are related to the doses applied and the method of application, since in practice, overdosing is sometimes used to achieve greater success in weed control. Additionally, localized applications tend to force a higher deposition of the herbicide at the same spot in the area, leading to a substantial increase in the recommended dose. In these situations, there will be a longer residual effect of picloram, making it necessary to wait an even longer period before planting susceptible crops such as soybeans.

## 3 Damage Caused by Picloram

Regarding the symptoms observed as a result of the injuries caused by picloram, it is noteworthy that the symptoms are usually more pronounced in broadleaf crops, since economically important narrow-leaved species tend to be slightly more tolerant to the presence of these residues in the soil.^15^ Specifically for soybean crops, among the main symptoms observed in plants grown in areas containing picloram residues in the soil are the occurrence of epinasty in the youngest trifoliate leaves, deformation of the leaf blade, growth arrest, and thickening of the roots (Figure 2). In the case of crops classified as narrow-leaved, in addition to some of the symptoms mentioned above, it is common to observe the formation of adventitious roots. Depending on the amount of picloram residue in the soil, this can lead to the death of sensitive crop plants, which happens gradually, occurring between 2 and 4 weeks after seedling emergence.

**Figure 2:**
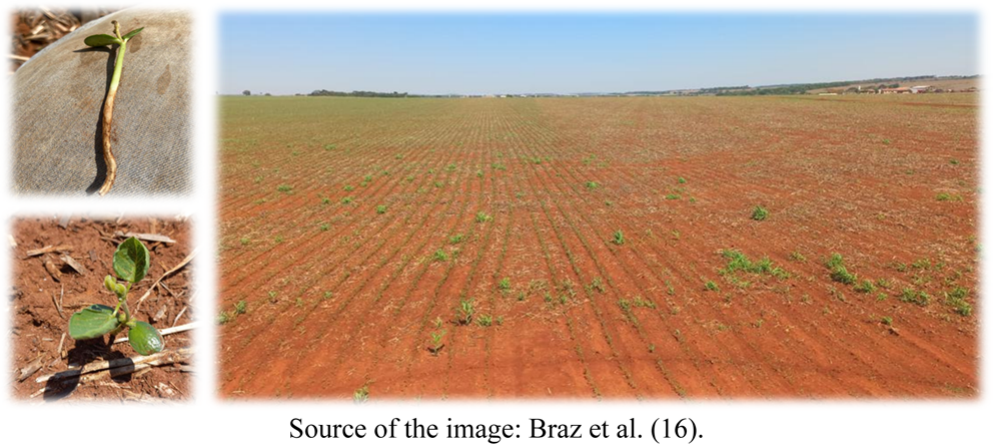
Injuries caused to soybeans by the presence of auxinic herbicide residues in the soil.

The losses resulting from injuries caused by the presence of picloram residues in soybeans will impact the morphophysiological development of the plants and, depending on the intensity, may result in the inability to produce grains in these areas. In cases of milder symptoms, due to lower concentrations of picloram in the soil, the losses will be related to gaps in crop stand, plants with smaller stature and leaf area index, and reduced photosynthetic capacity. Regardless of the scenario, if there are picloram residues in the soil available for plant absorption, losses in soybean yield will be observed. Just to illustrate soybean sensitivity to picloram, in a study conducted by Braz et al.^16^ aimed at correlating the concentration of this herbicide in the soil with the yield of different cultivars, it was observed that for every 0.05 mg a.i. kg^–1^ of soil, there was a reduction in crop yield of 6.4 bags per hectare (average of three soybean cultivars) (Figure 3).

**Figure 3:**
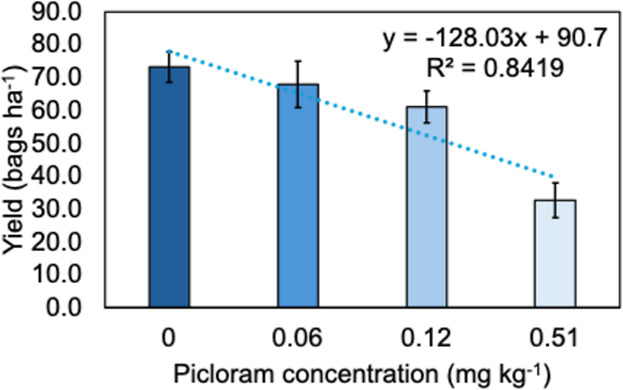
Correlation between picloram concentration in the soil and soybean grain yield in different cultivars.

In addition to the direct damages caused by the presence of picloram in the soil, another issue related to the use of this herbicide concerns the fact that, at times, this molecule is not degraded even after passing through the digestive tract of animals such as cattle.^17,18^ In this situation, the animal feeds on pasture in areas where picloram has been previously applied and, after the digestion process, there is still a possibility of the manure being contaminated, which prevents this residue from being used for agricultural purposes.

## 4 Methods for Detecting Picloram in Soil

Currently, there are different possibilities that can be employed with the aim of detecting whether a particular area contains picloram residue in the soil. Despite this, there are still bottlenecks in these methodologies, which ultimately limit the safe recommendation of when soybean cultivation can or cannot be carried out if picloram is detected. However, the integrated adoption of different detection methods may provide more accurate guidance in identifying whether an area presents a potential risk for soybean cultivation due to the presence of residues from this auxinic herbicide.

The first methodology that can be employed to determine whether a soil does or does not present a risk of having picloram residue involves analyzing the history of herbicide use over previous years. By keeping a historical record of the herbicides used in pasture areas, it becomes possible to identify which commercial products were applied, the dosages, the application methods, and the interval between the use of the herbicide and the potential time for soybean planting (Figure 4). Based on this information, it is possible to determine whether there is a risk associated with cultivating soybeans in each area. The main challenge in adopting this methodology lies in the producer’s ability to properly organize and store the complete history of herbicide applications in the pasture.

**Figure 4:**
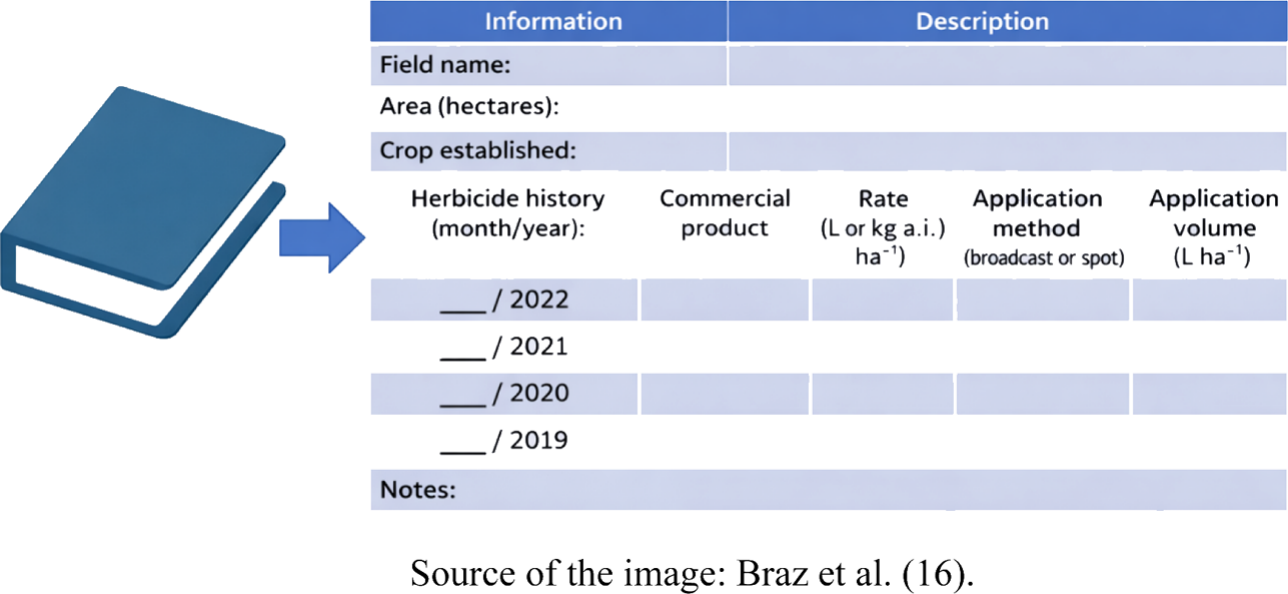
Field notebook template for recording the history of herbicide use in pasture areas.

Another methodology that can be used to identify picloram residues in the soil involves the use of bioindicator plants, which must be highly sensitive to the active ingredient. This sensitivity allows the visualization of injuries caused by the herbicide’s action even at very low doses. For picloram, alternative species that can be used in bioassays include tomato, cucumber, beetroot,^19^ beans, and soybean itself.^5^ Among the advantages of adopting this detection methodology are its low cost, since only the purchase of seeds of the bioindicator species is needed, and the speed in obtaining results, as injuries resulting from the toxic action of picloram usually appear a few days after seedling emergence.^15,19^

In contrast, the use of bioassays still presents some bottlenecks, such as the inability to accurately quantify the concentration of picloram in the soil, since it is only a qualitative method that determines whether the herbicide is present or not. Another obstacle regarding the use of bioindicator species is that the results will demonstrate the specific behavior for the area where the bioassay was conducted, making it impossible to extrapolate the findings to the entire plot. Considering that commercial products based on picloram are sometimes applied in localized areas, the bioassay methodology may not be able to identify specific patches with the presence of the herbicide if the sampled area does not coincide with the area that has a history of application.

Finally, in recent harvests, a methodology that has been used for detecting picloram residues in the soil is the technique of liquid or gas chromatography. In this analytical procedure, it is possible to quantify the presence of picloram in the soil. To increase the accuracy of this methodology, it is essential to ensure that soil sampling is carried out according to the laboratory’s technical recommendations, since any error in this procedure may lead to misinterpretation.

Another important point regarding the use of chromatography for measuring picloram residues in the soil concerns the challenges in the analytical interpretation of samples, since there are situations where the analysis detects the presence of the herbicide (reading > than the detection limit), but the concentration is below the quantification limit. In this scenario, it is not possible to be certain about the viability of soybean cultivation in the area. Currently, there is no established literature or practical guideline indicating the critical level of picloram in the soil (concentration) based on chromatographic analyses above which soybean cultivation would not be recommended. Significant advances have been made in methods for quantifying picloram using liquid chromatography coupled to mass spectrometry (LC–MS/MS), allowing the detection of low picogram (pg) concentrations of this compound in the column.^20^ In addition, Scutariu et al.^21^ describe a quantitative method using LC–MS/MS that provided high quantification limits for picloram (0.51 ng/g in soil samples and 0.74 ng/g in plant samples).^21^ Other challenges in using chromatographic analyses include the high cost of processing a sample, as well as the search for laboratories that perform this type of analysis, since this technique is not yet fully widespread in different regions of Brazil.

Additionally, although this review focuses on the detection of picloram residues in soil, and the limitations of current analytical methods have been pointed out, there are also limitations regarding the detection of herbicides in plants. Studies aimed at detecting auxin herbicides in plants have already demonstrated that the timing of plant sampling influences the quantification of these active ingredients in plant tissues.^22^ Summarizing the information regarding detection methodologies, although further advancements are still needed for a confident assessment of soybean cultivation potential, recording the application history of auxinic herbicides, using bioindicator species, and conducting chromatographic analyses may, together, provide a more accurate estimate of the necessary interval for the safe establishment of soybean in areas with a history of commercial products based on picloram.

## 5 Alternatives to Mitigate Losses

After demonstrating the impacts caused by the presence of picloram residues and the methodologies that can be used to detect this molecule in the soil, the final section of this review aims to present some alternatives that can be employed to mitigate the damages resulting from the use of this herbicide in livestock areas prior to soybean cultivation. As a first step, it is suggested that before using picloram-based products, a thorough analysis should be conducted regarding the possibility of transitioning the area from livestock to grain production. Based on this analysis, if the livestock farmer intends to lease the property for soybean cultivation, it is recommended to avoid the use of these commercial products to prevent issues related to residues. To prevent soil contamination with picloram residues, it is essential to prioritize the rational use of commercial products containing this active ingredient, making substitutions and/or rotations with herbicide molecules that have a shorter residual activity in the soil (such as triclopyr, fluoroxypyr, and 2,4-D amine).^10^

In situations where the herbicide has already been previously applied, studies have shown the potential use of certain plants to decontaminate soil with picloram, in a process known as phytoremediation.^6,23^ The phytoremediation process consists of using plant species that can absorb contaminants present in each environment (e.g.: soil, water, or air), and through this removal, remediate problems related to the presence of the compound.^24^ Currently, studies conducted in Brazil have already identified, through the selection of plants that tolerate the presence of picloram in the soil, the species that show the greatest tolerance to this active ingredient.^15^ Among the plants that showed the greatest suitability for use in the remediation process, two species from the Poaceae family stood out: *Eleusine coracana* and *Panicum maximum*.

Studies conducted with these species demonstrate that the remediation process using the plants *E. coracana* and *P. maximum* is influenced by the extension of cultivation period, plant density, soil water availability, herbicide applied dose, and the physical-chemical properties of the soil.^25,26^ Although this practice shows promise, the research is still preliminary, and further development is needed before it can be recommended at the field level. In this context, one of the limitations identified for the use of plants that showed phytoremediation potential (*E. coracana* and *P. maximum*) was the potential to recontaminate the soil after the extraction process.^27^ Chromatographic analyses revealed that, after absorption, the picloram molecule remains herbicidally active, indicating that there is a limited metabolic conversion of the active ingredient into nontoxic compounds.^27^

Due to the potential use of phytoremediation in soils contaminated with picloram, studies aimed at amplifying this process are important. In this context, the use of microorganisms presents in the soil that act in the picloram degradation process may be an interesting approach to be used in an integrated way with remediating plant species.^28^ Another aspect that influences the dissipation process of picloram in the environment relates to soil pH, a scenario in which it has been observed that soils with pH values closer to neutrality tend to have greater leaching of the active ingredient.^12^ In this sense, in soils contaminated with picloram, care must be taken with liming, since this practice will directly influence the dynamics of picloram in the environment.

Another option for areas with a history of picloram application is to adopt crop rotation schemes, introducing corn and sorghum, as these crops show greater tolerance to the presence of this molecule’s residues in the soil.^5^ Although these cereals are less profitable than soybeans, growing corn and sorghum offers the possibility of some profit in scenarios where growing oilseeds is not technically viable. In addition, for the areas where the presence of picloram residues was detected but the concentrations were low (values < detection limit), one possibility being studied concerns the use of cultivars with differential tolerance in terms of sensitivity to the herbicide (Figure 2). Although all cultivars are known to be sensitive to the presence of picloram residues in the soil, in situations where this herbicide is present at very low concentrations, there may apparently be different responses among soybean genetic materials.^16^ Currently, there is still no clear answer regarding the effect on the behavior of soybean cultivars, but it appears that materials with a higher maturity group (later maturity) tend to show less reduction in grain yield because of the phytotoxic effects caused by the presence of picloram residues in the soil at lower concentrations.

## 6 Final Considerations

In the context of increasing soybean cultivation areas projected for upcoming harvests in regions traditionally used for livestock farming, there is a possibility of a higher frequency of problems arising from crop intoxication due to the presence of picloram in the soil. Among the main damages observed in soybeans caused by the presence of this molecule in the soil environment are reduced plant stands, decreased plant size and leaf area, and lower grain yield. In some cases, depending on the concentrations of picloram in the soil, oilseed production may even become unfeasible. Therefore, it is essential to adopt detection methods (usage history, bioassays, and chromatographic analysis), which together can provide greater predictability regarding the feasibility of soybean cultivation in areas with a history of picloram application. To mitigate the damage caused by picloram, it is recommended that the use of this herbicide in areas intended for animal production be carried out rationally, and that crop rotation schemes with species more tolerant to the herbicide (e.g., corn and sorghum) be implemented.
